# *Aeromonas caviae* subsp. *aquatica* subsp. nov., a New Multidrug-Resistant Subspecies Isolated from a Drinking Water Storage Tank

**DOI:** 10.3390/microorganisms13040897

**Published:** 2025-04-13

**Authors:** Victor Hugo Moreira, Lidiane Coelho Berbert, Ayodele Timilehin Adesoji, Kayo Bianco, Janaina Japiassu Vasconcelos Cavalcante, Flávia Lúcia Piffano Costa Pellegrino, Rodolpho Mattos Albano, Maysa Mandetta Clementino, Alexander Machado Cardoso

**Affiliations:** 1 Department of Biology, Rio de Janeiro State University, Rio de Janeiro 20550-013, Brazil; vhm.18@hotmail.com (V.H.M.); lidy_berbert@hotmail.com (L.C.B.); 2 Department of Microbiology, Federal University, Dutsin-Ma 821101, Nigeria; timmyayus2002@yahoo.com; 3 National Institute for Quality Control in Health, Oswaldo Cruz Foundation, Rio de Janeiro 21040-900, Brazil; kayo.bianco@fiocruz.br (K.B.); maysa.mandetta@fiocruz.br (M.M.C.); 4 Division of Metrology Applied to Life Sciences, National Institute of Metrology, Quality and Technology, Duque de Caxias 25212-435, Brazil; cavalcantejanaina@yahoo.com.br; 5 Department of Pharmacy, Rio de Janeiro State University, Rio de Janeiro 20550-013, Brazil; flpellegrino@hotmail.com; 6 Department of Biochemistry, Rio de Janeiro State University, Rio de Janeiro 20550-013, Brazil; albano@uerj.br

**Keywords:** *Aeromonas*, antibiotics, chlorine, water distribution systems, drinking water

## Abstract

The increasing prevalence and dissemination of multidrug-resistant bacteria represent a serious concern for public health. *Aeromonas caviae* is a pathogenic microorganism that causes a wide spectrum of diseases in fish and humans and is often associated with aquatic environments and isolated from foods and animals. Here, we present the isolation and characterization of the V15^T^ strain isolated from a drinking water storage tank in Rio de Janeiro, Brazil. The V15^T^ strain has a genome length of 4,443,347 bp with an average G + C content of 61.78% and a total of 4028 open reading frames. Its genome harbors eight types of antibiotic resistance genes (ARGs) involving resistance to beta-lactamases, macrolides, and quinolones. The presence of *bla_MOX-6_*, *bla_OXA-427_/bla_OXA-504_*, and mutations in *parC* were detected. In addition, other ARGs (*macA*, *macB*, *opmH*, and *qnrA*) and multidrug efflux pumps (such as *MdtL*), along with several resistance determinants and 106 genes encoding virulence factors, including adherence (polar and lateral flagella), secretion (T2SS, T6SS), toxin (*hlyA*), and stress adaptation (*katG*) systems, were observed. The genome sequence reported here provides insights into antibiotic resistance, biofilm formation, evolution, and virulence in *Aeromonas* strains, highlighting the need for more public health attention and the further monitoring of drinking water systems. Also, the results of physiological and phylogenetic data, average nucleotide identity (ANI) calculation, and digital DNA–DNA hybridization (dDDH) analysis support the inclusion of the strain V15^T^ in the genus *Aeromonas* as a new subspecies with the proposed name *Aeromonas caviae* subsp. *aquatica* subsp. nov. (V15^T^ = P53320^T^). This study highlights the genomic plasticity and pathogenic potential of *Aeromonas* within household drinking water systems, calling for the revision of water treatment protocols to address biofilm-mediated resistance and the implementation of routine genomic surveillance to mitigate public health risks.

## 1. Introduction

Access to clean drinking water is a fundamental human right, yet microbial contamination remains a persistent concern worldwide [1]. *Aeromonas* spp. are ubiquitous in aquatic environments and are frequently detected in treated drinking water [2]. While often considered opportunistic pathogens, certain strains have been linked to severe infections in immunocompromised individuals [3]. The ability of *Aeromonas* to persist in drinking water distribution systems, often forming biofilms and acquiring antibiotic resistance genes (ARGs), poses significant challenges to water quality management [4].

Filtration followed by chlorine disinfection has been used for water purification before human consumption for over 100 years. The adoption of this practice drastically reduced the cases of waterborne diseases associated with contaminated water. Wastewater treatment plants employ a series of procedures to remove contaminants and pathogenic microorganisms from untreated water to obtain safe drinking water to prevent diseases in the final consumers [5,6].

In recent years, there has been increasing concern about pathogens capable of resisting conventional water treatment and surviving in water distribution systems. These microorganisms can reach the final consumers, bringing serious health risks [7]. These organisms are characterized by being residents of the entire water treatment system, being adapted to the oligotrophic conditions of the treated water, and being able to proliferate in tanks designed for the bulk storage of potable drinking water. In addition, many of these organisms are considered opportunistic, as they cause infections in children, the elderly, and immunocompromised patients. Among these microorganisms, *Aeromonas* spp. stand out [8,9,10].

*Aeromonas caviae* is a facultative anaerobe and rod-shaped Gram-negative bacterium, often found in different aquatic environments, including chlorinated drinking water [7,11]. Even in developed countries where appropriate sanitary conditions are carefully monitored and enforced, *Aeromonas* represents a threat. A comprehensive survey in the United States demonstrated that exposures to aeromonads in drinking water supplies are widespread [2].

The global incidence of *Aeromonas* infection remains underreported; however, regional studies suggest that the variability in infection rates may reflect disparities in public health infrastructure, the climate, environmental exposure, and socioeconomic conditions [3,12,13,14,15,16]. While some regions maintain stable incidence rates, factors such as seasonal flooding, recreational water exposure, climate change, increasing antimicrobial resistance, and population growth may alter epidemiological patterns [3,12,13]. Rising temperatures have been linked to increased antibiotic resistance, which complicates the treatment of bacterial infections, including those caused by *Aeromonas*. This resistance can spread more rapidly in environments with inadequate public health infrastructure [14,15].

The pathogenicity of *Aeromonas* is usually considered to be multifactorial as several antibiotic resistance genes and virulence factors have been identified, and metabolic adaptations may be associated with survival in different habitats [11,17]. Species of the genus *Aeromonas* are recognized pathogens in a broad range of aquatic animals, often contributing to disease outbreaks in aquaculture systems. In humans, *Aeromonas* infections are primarily associated with gastroenteritis and soft tissue infections, particularly in immunocompromised individuals or following exposure to contaminated water sources [18,19].

Human infections caused by *Aeromonas* spp., particularly *A. caviae* and *A. veronii*, are increasingly recognized in clinical settings, with manifestations ranging from gastroenteritis to life-threatening systemic diseases [20]. Gastrointestinal illness is primarily associated with the ingestion of contaminated water or food, especially in regions with inadequate sanitation infrastructure. *A. caviae* is frequently implicated in diarrheal disease, with clinical presentations ranging from self-limiting watery diarrhea to dysentery-like syndromes. In severe cases, symptoms may resemble cholera, leading to profound dehydration and electrolyte imbalances [21]. The pathogenic potential of clinical strains has been attributed to their capacity to adhere to and invade intestinal epithelial cells, resulting in microvilli disruption and compromised mucosal integrity. Soft tissue and wound infections typically arise following direct exposure to contaminated water, particularly after traumatic injuries such as fishing-related cuts or burns [22]. Immunocompromised individuals, such as those with chronic liver disease, malignancies, or who are undergoing immunosuppressive therapy, are especially susceptible. In these patients, *Aeromonas* infections can rapidly progress to necrotizing fasciitis, septicemia, and multiorgan failure [18,19,20,21,22].

Emerging evidence underscores the potential of *Aeromonas* spp. as sentinel organisms within a One Health framework for monitoring antimicrobial resistance (AMR) across interconnected human, animal, and environmental domains [23,24]. Recent meta-analyses have highlighted the frequent detection of multidrug-resistant *Aeromonas* strains, including extended-spectrum β-lactamase (ESBL) producers, in treated and post-chlorinated wastewater effluents [24,25,26]. These findings raise significant public health concerns, particularly given the genus’s ubiquity in aquatic ecosystems and its capacity to serve as a reservoir for clinically relevant resistance genes. Antibiotic-resistant species are a challenge for the treatment of infections, and water has been considered a possible reservoir of resistance genes [27,28].

Despite ongoing advances in infrastructure, access to a reliable water supply remains sporadic across vast regions of Latin America, Asia, and Africa. In response to these systemic shortcomings, local populations have adapted by employing diverse strategies for the collection, transport, and domestic storage of water. Here, we report the isolation and characterization of a novel subspecies, *Aeromonas caviae* subsp. *aquatica* subsp. nov., isolated from a domestic drinking water storage tank in Brazil, to obtain insights into the molecular mechanisms of antibiotic resistance and virulence factors that could potentially pose a threat to human health.

## 2. Materials and Methods

### 2.1. Sample Collection

Water samples were collected from the plastic storage containers of urban residents (n = 25) located in Rio de Janeiro, Brazil (22.919 S, 43.684 W), monthly during summer (December to March). The samples were collected in 5 L sterile glass bottles, transported refrigerated to the laboratory, and 2 L from each collection point were concentrated through filtration through 0.22 μM cellulose membranes (Millipore, Burlington, MA, USA). The membranes were inoculated in an alkaline peptone water broth (APW) and incubated at 37 °C for 24 h. The culture growth was seeded on an *Aeromonas* isolation agar—AIA (Sigma-Aldrich, St. Louis, MO, USA)—medium at 30 °C for 24 h. Approximately four colonies per sample with typical *Aeromonas* morphology (green colonies with dark end centers) were inoculated in tryptone soy agar—TSA (Sigma-Aldrich)—and incubated at 30 °C for 24 h for further identification as previously described [29].

### 2.2. Characterization and Identification of Isolates

Bacterial isolates were analyzed through biochemical tests measuring carbon source utilization, enzymatic activities, and antibiotic resistance using the VITEK2 identification system (BioMérieux, Marcy-l′Étoile, France) according to the manufacturer’s recommendations. The lipid profile was analyzed using gas chromatography–mass spectrometry (GC-MS) as described previously [30]. Briefly, lipids were extracted from bacterial cells using a chloroform–methanol mixture (2:1, *v*/*v*). The lipid profile was compared with those of other *Aeromonas* species to identify unique lipid signatures associated with the novel subspecies. The broth microdilution method VET04-A [31] was used to determine the minimum inhibitory concentration—MIC—of 14 antimicrobial agents for *Aeromonas*. Multiple-drug resistance (MDR) was defined as resistance to three or more antibiotic classes. We used 96-well microplates and performed serial dilutions of the antimicrobial solutions to obtain the final concentrations of cefotaxime (0.031–64 mg/L), ceftazidime (0.031–64 mg/L), chloramphenicol (0.062–64 mg/L), enrofloxacin (0.008–16 mg/L), erythromycin (0.031–64 mg/L), florfenicol (0.062–128 mg/L), flumequine (0.008–16 mg/L), gentamicin (0.062–64 mg/L), nalidixic acid (0.062–128 mg/L), oxolinic acid (0.008–16 mg/L), streptomycin (0.125–256 mg/L), temocillin (0.125–256 mg/L), tetracycline (0.062–128 mg/L), and trimethoprim–sulfamethoxazole (0.031/0.589–8/152 mg/L) as described previously [32]. To confirm the suggestive *Aeromonas* spp., 16S rRNA gene amplification and sequencing were carried out using the primers AERF, 5′-CTACTTTTGCCGGCGAGCGG-3′, and AERR, 5′-TGATTCCCGAAGGCACTCCC-3′ [33].

### 2.3. Genome Sequencing and Bioinformatic Analysis

Genomic DNA was extracted from a pure culture using the GenElute Bacterial Genomic DNA kit (Sigma-Aldrich, USA). Whole-genome sequencing (WGS) was performed using the Nextera XT DNA Sample Preparation kit (Illumina, San Diego, CA, USA) and the Illumina MiSeq platform according to the standard operation based on a paired-end library. Genome assembly was performed with A5 miseq software (Version 3.0) [34]. The genome completeness was checked using checkM v1.0.11 [35]. The genome sequence data were uploaded to the Type (Strain) Genome Server (TYGS) for a whole-genome-based taxonomic analysis [36] to perform a digital DNA-DNA hybridization (dDDH) analysis. The Average Nucleotide Identity (ANI) between bacterial genomes was assessed using the EzBioCloud orthoANI (Orthologous Average Nucleotide Identity) tool [37]. The *A. caviae* V15^T^ genome was annotated with Rapid Annotation using Subsystem Technology, RAST [38], and the Pathosystems Resource Integration Center (PATRIC) genome annotation tool [39]. The existence of plasmids was assessed using PlasmidFinder [40]. Antimicrobial resistance (AMR) genes and virulence factors were also identified by using the Resfinder database and the Kyoto Encyclopedia of Genes and Genomes database (KEGG) Pathway database.

### 2.4. Genome Sequence Data Availability

This Whole-Genome Shotgun project has been deposited at DDBJ/ENA/GenBank under the accession number JAOVTG000000000. The version described in this paper is version JAOVTG010000000.

## 3. Results and Discussion

### 3.1. Characterization of Isolates

A total of 101 possible *Aeromonas* colonies, based on morphological characters in the AIA medium, were isolated from twenty-five water samples from the plastic storage containers (known locally as ‘caixas d’água’) of urban residents in Rio de Janeiro, Brazil, as previously described [30]. These tanks are standard in Brazilian homes, as most residences utilize storage systems to compensate for an intermittent public water supply. While the source water is treated municipal tap water (chlorinated; residual chlorine = 0.2–0.5 mg/L), it undergoes subsequent storage in these tanks for periods ranging from 24 h to several months. Current Brazilian sanitation regulations recommend but do not mandate the cleaning of these tanks every six months. However, compliance is variable, and there is no systematic monitoring or enforcement of these guidelines [41]. This lack of standardized maintenance creates potential reservoirs for biofilm formation and bacterial proliferation, particularly for chlorine-resistant organisms like *Aeromonas* spp.

The biochemical tests showed that 42.57% (43/101) of isolates were Gram-negative, rod-shaped and facultative, and oxidase-, catalase-, lysine decarboxylase-, and indole-positive, all suggestive of the *Aeromonas* genus. Then, the confirmation of putative *Aeromonas* spp. was performed by PCR and sequencing [33]. The susceptibility to antimicrobials was also analyzed using the VITEK2 Compact system. It was observed that 86.49% (32/37) of the isolates were classified as having an MDR profile and 5 as non-MDR, being resistant only to ampicillin (Table 1). The MIC distributions for amikacin, gentamicin, and piperacilin/tazobactam of the isolates were similar to those for *Aeromonas caviae* from multiple sources, geographical areas, and time periods [42,43]. The *Aeromonas caviae* strain V15 exhibited a multidrug-resistant (MDR) phenotype, characterized by high-level resistance to β-lactams, including ampicillin (AMP; MIC ≥ 64), ceftazidime (CAZ; MIC ≥ 64), aztreonam (ATM; MIC 32), and carbapenems (imipenem, IPM, MIC of 8; meropenem, MEM, MIC ≥ 16), as well as fluoroquinolones (ciprofloxacin, CIP; MIC ≥ 4) and trimethoprim–sulfamethoxazole (SXT; MIC ≥ 150). Notably, V15 had a similar resistance profile to FAHZZU2447 [44], which also demonstrated resistance to CAZ (MIC ≥ 128), ATM (MIC 32), and IPM (MIC 8), suggesting the possible presence of extended-spectrum β-lactamases (ESBLs) or metallo-β-lactamases (MBLs). In contrast, strain 211703 [45] displayed a divergent susceptibility pattern, remaining sensitive to aztreonam (ATM; MIC ≤ 1) but resistant to carbapenems (IPM ≥ 16, MEM ≥ 16) and fluoroquinolones (CIP ≥ 4), indicating a different underlying resistance mechanism, potentially involving carbapenemase production without concomitant ATM resistance. All three strains retained susceptibility to amikacin (AMK) and tigecycline (TGC), underscoring these agents as viable therapeutic options (Table 1).

The biochemical profiling of strain V15^T^ revealed positive activity for several enzymatic and metabolic substrates, including ala-phe-pro-arylamidase (APPA), D-cellobiose (dCEL), beta-galactosidase (BGAL), B-N-acetylglucosaminidase (BNAG), D-glucose (dGLU), glucose fermentation (OFF), beta-glucosidase (BGLU), D-maltose (dMAL), D-mannitol (dMAN), L-proline arylamidase (ProA), Lipase (LIP), tyrosine arylamidase (TyrA), Saccharose/Sucrose (SAC), D-trehalose (dTRE), L-lactate alkalinization (LATk), and Succinate alkalinization (SUCT), and negative activity for Adonitol (ADO), L-pyrrolydonyl-arylamidase (PyrA), L-arabitol (IARL), H2S production (H2S), glutamyl arylamidase pNA (AGLTp), gama-glutamyl-transferase (GGT), D-mannose (dMNE), beta-xylosidase (BXYL), beta-alanine acrylamidase (BAlap), Palatinose (PLE), Urease (URE), D-sorbytol (dSOR), D-tagatose (dTAG), Citrate Sodium (CIT), Malonate (MNT), 5-keto-D-gluconate (5KG), alpha-glucosidase (AGLU), B-N-acetylgalactosaminidase (NAGA), alpha-galactosidase (AGAL), Phosphatase (PHOS), glycine arylamidase (GlyA), and ornithine decarboxylase (ODC). To further elucidate the genomic potential of this multidrug-resistant strain, a whole-genome sequencing analysis was performed.

### 3.2. Genomic and Lipid Profiles

**Figure 1:**
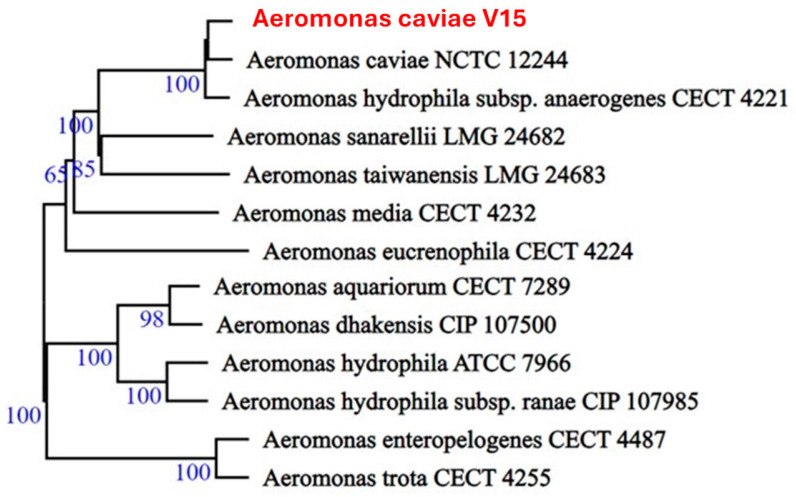
Phylogenetic tree inferred from GBDP distances calculated from genome sequences. Numbers above branches indicate GBDP pseudo-bootstrap support values (>60%) based on 100 replicates, with average branch support of 90.1%.

The *Aeromonas caviae* V15^T^ genome assembly did not show any contaminants and achieved 99.97% completeness as shown through CheckM analysis. In silico plasmid detection and typing revealed no plasmids in this strain. The genome consisted of 35 contigs with a total length of 4,443,347 bp and an average G + C content of 61.78%. Both the size and G + C content were similar to data published for other *Aeromonas* genomes (Table 2). In silico DDH (dDDH) analysis demonstrated that the *Aeromonas caviae* NCTC 12244 reference genome was the most similar to the V15^T^ strain, with a dDDH of 91.7%. ANI analysis resulted in a score of 98.14% for the type strain *Aeromonas caviae* 8LM isolated from a stool culture from a child with diarrhea in southern Brazil [49]. The ANI and dDDH analyses strongly indicated that the V15^T^ strain belongs to the *A. caviae* species cluster. The Genome BLAST (Version 3.0) Distance Phylogeny approach (GBDP) revealed similar results (Figure 1). Moreover, the assembled genome was submitted to the *Aeromonas* MLST (multi-locus sequence typing) database, which assessed the allelic profiles of six (*gltA*, *groL*, *gyrB*, *metG*, *ppsA*, and *recA*) housekeeping genes [50]; the MLST typing method proved that V15^T^ belongs to a new sequence type (ST), ST2749.

In addition, the lipid profile of *Aeromonas caviae* subsp. *aquatica* subsp. nov. revealed the presence of several fatty acids commonly found in *Aeromonas* species, including palmitic acid (C16:0), palmitoleic acid (C16:1), stearic acid (C18:0), oleic acid (C18:1), and linoleic acid (C18:2) [51]. Moreover, the strain exhibited a unique lipid signature characterized by the presence of branched-chain fatty acids (BCFAs) such as iso-C15:0 and anteiso-C15:0, which are known to play a role in membrane fluidity and stress tolerance [52]. The presence of these BCFAs may contribute to the strain’s ability to survive in oligotrophic environments, such as drinking water storage tanks. The lipid profile analysis also identified the presence of phosphatidylethanolamine (PE) and phosphatidylglycerol (PG), which are major phospholipids in the bacterial membrane and are essential for maintaining membrane integrity and function [53].

**Figure 2:**
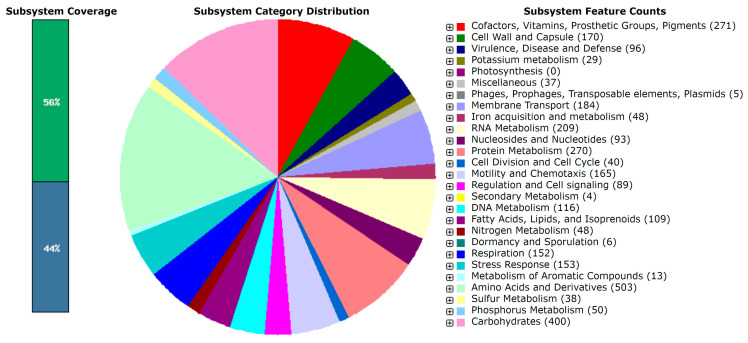
Subsystem category distribution for *Aeromonas caviae* V15^T^ determined using the RAST server [26]. The pie chart shows the count of each subsystem feature and the subsystem coverage.

The annotation of the genome of V15^T^ with Rapid Annotation using Subsystem Technology (RAST) identified 4028 putative coding sequences (CDSs) and 147 RNAs. An overview of the count of each feature in the subsystem and its coverage is shown in Figure 2.

The subsystem annotation of the *Aeromonas caviae* V15^T^ genome revealed a diverse functional profile, with approximately 56% of the genome assigned to known subsystems and 44% remaining unclassified. This level of subsystem coverage is comparable to that of previously sequenced *Aeromonas* strains, such as *Aeromonas hydrophila* ATCC 7966, which also showed a significant portion of genes with unknown functions, indicating the genetic complexity of the genus [54].

A substantial proportion of the annotated genes in *A. caviae* V15^T^ are involved in core metabolic pathways, including carbohydrate metabolism (400 features), amino acid and derivative metabolism (503 features), and protein metabolism (270 features). These numbers are consistent with those of other *Aeromonas* genomes, such as *Aeromonas veronii* B565, which has a similarly large proportion of genes dedicated to these essential functions [55]. The ability to efficiently metabolize carbohydrates and amino acids may contribute to the ecological versatility of *A. caviae* V15^T^, allowing it to thrive in various aquatic environments.

Regarding environmental adaptation, *A. caviae* V15^T^ exhibits genes associated with stress response (153 features) and dormancy/sporulation mechanisms (6 features). This genomic composition suggests resilience to fluctuating environmental conditions, similar to that of *Aeromonas salmonicida*, which has been reported to survive in oligotrophic and cold-water habitats due to similar stress response pathways [56]. The lipid metabolism of *A. caviae* V15^T^, indicated by the presence of 109 genes related to fatty acids, lipids, and isoprenoids, is noteworthy. Studies on *Aeromonas* species have shown that the lipid composition can influence membrane fluidity and stress resistance [3]. The detection of phosphatidylethanolamine and phosphatidylglycerol in *A. caviae* V15^T^ further supports its adaptation to diverse environmental conditions. Interestingly, the strain harbors only five genes related to phages, prophages, transposable elements, and plasmids, suggesting a relatively stable genome with limited horizontal gene transfer. In contrast, *A. hydrophila* ATCC 7966 has a higher number of mobile genetic elements, which may contribute to its greater genetic plasticity [54].

### 3.3. Antibiotic Resistance and Virulence Factors

Genome annotation revealed a repertoire of 172 genes or gene clusters putatively linked to virulence, disease, and defense mechanisms, including 93 genes associated with resistance to antibiotics and toxic compounds. Among these, multiple loci encoded resistance determinants for β-lactams, macrolides, quinolones, and multidrug efflux systems, reflecting a broad spectrum of antimicrobial defense. This genomic profile parallels those reported in *Aeromonas hydrophila* and *Aeromonas veronii*, which similarly harbor diverse virulence-associated genes implicated in their opportunistic pathogenicity in both humans and animals [57]. In addition, the genome harbored putative virulence factors potentially involved in host colonization and pathogenesis, including genes encoding hemolysin (*hlyA*), and motility- and adhesion-related proteins such as polar flagellar components, lateral flagella, and type IV pili. Secretory machinery components, notably from type II and type VI secretion systems, were also identified (Table S1), further supporting this organism’s potential for environmental persistence and host interaction.

In this study, we identified multiple genes conferring resistance to β-lactam antibiotics, including *cphA*, encoding a subclass B2 metallo-β-lactamase; *bla_MOX_*, encoding a class C β-lactamase; and *bla_OXA_*, encoding a class D β-lactamase. While β-lactamase genes are frequently associated with mobile genetic elements such as plasmids [58], transposases [59], and integrons [60], genomic context analysis revealed that none of these three loci were proximal to such elements. This spatial independence suggests that the production of β-lactamases in this strain may be intrinsic, in line with observations in other *Aeromonas* species [61]. These findings reinforce the potential for endogenous antibiotic resistance in environmental isolates and highlight the importance of genomic surveillance beyond the conventional focus on horizontal gene transfer.

Class B β-lactamases, also known as metallo-β-lactamases, are zinc-dependent enzymes that require divalent cations, typically Zn²⁺, as essential cofactors for catalytic activity. These enzymes exhibit hydrolytic activity against a broad range of β-lactam antibiotics, including carbapenems, and are capable of inactivating both penicillins and cephalosporins [62]. In *Aeromonas* spp., class B β-lactamases are considered intrinsic resistance determinants, with a conserved presence across multiple species such as *A. hydrophila*, *A. veronii*, and *A. dhakensis* [61]. Their widespread distribution within the genus underscores the evolutionary persistence of this resistance mechanism and its potential role in both environmental resilience and opportunistic pathogenicity. On the other hand, class C β-lactamases are serine-based enzymes with potent hydrolytic activity against cephalosporins and are notably resistant to inhibition by conventional β-lactamase inhibitors, including clavulanic acid, tazobactam, and sulbactam [63]. In the present study, we identified the *bla_MOX_* gene, which encodes an AmpC-type class C β-lactamase. This gene has previously been reported in *Aeromonas caviae* strains isolated from patients with bacteremia, where it contributes to antibiotic resistance phenotypes [64]. Clinically, *bla_MOX_*-like genes have been implicated in reduced susceptibility to third-generation cephalosporins such as ceftriaxone, which remains a standard therapeutic agent for *Aeromonas*-associated gastroenteritis [65]. Additionally, class D β-lactamases, or oxacillinases, display strong hydrolytic activity against oxacillin and can confer resistance to a broader spectrum of β-lactams, including penicillins, temocillin, ceftazidime, and aztreonam [66]. The co-occurrence of class C and D β-lactamases within a single strain underscores the multifaceted resistance potential of *Aeromonas* spp. and highlights the clinical relevance of continued genomic surveillance.

In the V15^T^ strain, we identified the *opmH* gene, which encodes the outer membrane channel TolC, alongside *macA* and *macB*, which encode the membrane fusion protein MacA and the ATP-binding cassette (ABC) transporter MacB, respectively. Together, these components constitute the MacAB-TolC efflux complex, a macrolide-specific ABC-type transporter known to mediate the active export of macrolide antibiotics [67]. While the functional role of the MacAB-TolC system in *Aeromonas caviae* remains incompletely characterized, studies in *Aeromonas salmonicida* suggest that this efflux complex may contribute to the organism’s intermediate susceptibility to erythromycin, indicating a potential role in modulating macrolide resistance [68]. The presence of this system in V15^T^ underscores the importance of efflux-mediated mechanisms in the intrinsic resistance repertoire of *Aeromonas* spp., particularly in environmental strains with potential clinical relevance.

The genomic analysis of the V15^T^ strain revealed mutations in the *parC* gene, which encodes the A subunit of topoisomerase IV, as well as the presence of the *qnrB* gene, a plasmid-mediated quinolone resistance determinant. Specifically, two well-characterized amino acid substitutions were identified in ParC: Ser80→Ile and Glu84→Lys. These mutations have previously been reported in *Aeromonas caviae* and are associated with reduced susceptibility to fluoroquinolones, including nalidixic acid, ciprofloxacin, and norfloxacin [58,69,70]. The *qnrB* gene encodes a pentapeptide repeat protein that binds and protects both DNA gyrase and topoisomerase IV from quinolone inhibition, thereby conferring an additional layer of resistance [69]. Widely distributed across all continents, *qnrB* has been predominantly identified in clinical isolates of *Enterobacteriaceae* [71], where it is frequently associated with mobile genetic elements, including plasmids, transposons, and class 1 integrons [72]. Interestingly, in the V15^T^ genome, *qnrB* was found to be chromosomally encoded, suggesting a stable, vertically inherited resistance mechanism rather than one acquired via horizontal gene transfer. This chromosomal localization may reflect evolutionary adaptation to persistent environmental antibiotic pressure.

The V15^T^ genome encodes a wide array of virulence-associated genes, including those implicated in adhesion, motility, toxin production, and secretion systems (Table 3 and Table S1). V15^T^ exhibits a more comprehensive T6SS arsenal, including genes such as *atsDGHIJKLPQS*, *clpV1*, *dotU*, *hcp*, *vasHK/atsR*, *vgrG2G3*, and *vipAB*, compared to the limited presence of *atsD* in strain FAHZZU2447, suggesting a potentially enhanced capacity for interbacterial competition and host interaction [44]. Among the adhesion-related genes analyzed, the *Aeromonas* strain V15 exhibited a repertoire of 130 identifiable genes, surpassing *A. hydrophila* ATCC 7966 (113 genes), *A. veronii* B565 (111 genes), *and A. hydrophila* ML09-119 (113 genes), while only slightly trailing behind *A. salmonicida* A449, which harbored 146 genes. This genetic richness suggests that V15^T^ possesses a diverse arsenal of adhesion factors, potentially enhancing its ability to colonize host tissues or abiotic surfaces, such as drinking water storage tanks. The high degree of similarity with *A. hydrophila* ATCC 7966, a well-established reference strain in virulence studies, further underscores the relevance of V15^T^ as a promising model for functional and pathogenic investigations within the Aeromonas genus (Table S1).

Bacterial adhesion to host surfaces is a critical early step in colonization and infection. In strain V15^T^, adhesion-related structures included type IV pili, as well as polar and lateral flagella. Notably, we identified genes associated with the biogenesis of the mannose-sensitive hemagglutinin (*Msh*) pilus system, which is organized as a single operon, a configuration conserved across several *Aeromonas* species [73]. The Msh pilus has been demonstrated to mediate bacterial adhesion to host tissues and is also involved in biofilm formation. The experimental inactivation of this gene cluster in *Aeromonas* has been shown to reduce adherence to epithelial cells by more than tenfold, concomitant with a marked decline in biofilm development [73]. These findings underscore the functional importance of the Msh system in virulence and surface persistence, particularly in environmental strains with potential pathogenicity.

The Tap pilus system, encoded by a single gene cluster (tap, tpp), was also identified in the V15^T^ genome. This genetic organization mirrors that reported in *Aeromonas hydrophila* and *Aeromonas veronii* [74]. Although the disruption of the *tapABCD* cluster does not appear to impair bacterial adhesion to host cells, the *tapD* gene plays a critical role in the maturation of type II secretion system (T2SS) pseudopilins. The inactivation of *tapD* has been shown to significantly reduce the secretion of key virulence factors exported via the T2SS, including proteases, hemolysins, and DNases [75]. These findings suggest that while the Tap system may not directly mediate adherence, it contributes to the pathogenic potential of *Aeromonas* by enabling the extracellular export of effector molecules essential for host tissue invasion and immune evasion.

The V15^T^ genome encodes a complete polar and lateral flagellar system. This motility apparatus has been implicated in bacterial adhesion to host cells and in the development of biofilms. The experimental inactivation of genes involved in lateral flagellum biogenesis has been shown to significantly impair both motility and adhesion [76]. In addition, the polar flagellar system of *Aeromonas caviae* V15^T^ closely resembles that of *A. hydrophila*, with more than 50 genes distributed across five distinct chromosomal regions. The polar flagellum is known to contribute to multiple facets of pathogenesis, including surface adherence, biofilm maturation, and mucosal invasion [77]. The strain’s capacity for biofilm formation was further confirmed using the tube-based crystal violet staining method [78]. Collectively, these findings underscore the coordinated role of dual flagellar systems in mediating environmental persistence and host colonization.

Members of the genus *Aeromonas* are known to produce a diverse arsenal of exotoxins that contribute to their pathogenic potential. In the V15^T^ strain, genomic analysis revealed the presence of genes encoding the hemolysin *HlyA*. The *HlyA* hemolysin, originally characterized in *Aeromonas dhakensis*, exhibits demonstrable hemolytic activity, although its precise role in pathogenesis remains to be fully elucidated [79,80]. The concurrent presence of multiple exotoxin genes suggests a multifactorial virulence strategy that may enhance the ability of V15^T^ to colonize and damage host tissues. *Aeromonas* species secrete a wide array of extracellular enzymes that, although not directly involved in host infection, play critical roles in environmental adaptation [17]. In the V15^T^ strain, genes encoding enzymes such as RNase R, enolase, and siderophore biosynthesis proteins were identified. Notably, the *vacB* gene encodes RNase R, a cold-shock-associated exoribonuclease that facilitates bacterial growth under low-temperature conditions. Beyond its role in stress adaptation, vacB has been linked to virulence; mutants deficient in *vacB* exhibit impaired motility and a consequent reduction in their virulence potential [80]. These findings underscore the ecological versatility of *Aeromonas* and highlight the intersection between environmental fitness and the pathogenic capacity.

The *eno* gene, which encodes the glycolytic enzyme enolase, was identified in the V15^T^ genome. In *Aeromonas dhakensis*, enolase is known to be surface-expressed in isolates associated with diarrheal disease, suggesting a dual role in metabolism and virulence [81]. Functionally, enolase facilitates host tissue invasion by binding to plasminogen and promoting its conversion to plasmin, a proteolytic enzyme that degrades extracellular matrix components. This activity not only enables bacterial dissemination within host tissues but may also support nutrient acquisition during infection. Iron acquisition is a critical determinant of bacterial survival and virulence within the host environment. In strain V15^T^, multiple genes associated with iron uptake via siderophore-mediated pathways were identified. This mechanism is tightly regulated by the fur gene, a global regulator of iron homeostasis [81].

Additionally, the V15^T^ genome harbored gene clusters encoding components of the type II (T2SS) and type VI (T6SS) secretion systems (Table S1), both of which are implicated in the transport of toxins and effector proteins across bacterial membranes. These systems likely contribute to the pathogenic potential of the strain through the coordinated secretion of virulence factors and interbacterial competition [82]. In strain V15^T^, the type II secretion system (T2SS) is encoded within a single genomic cluster that constitutes the conserved core machinery. The T2SS is responsible for the extracellular export of a suite of pathogenic enzymes, including aerolysins, phospholipases, proteases, and DNases, that facilitate host tissue degradation and immune evasion [77]. The type VI secretion system (T6SS), also encoded within a gene cluster, plays a complementary role in virulence. Functional studies have shown that T6SS inactivation leads to diminished antiphagocytic activity and attenuated pathogenicity in murine models. In *Aeromonas dhakensis*, the T6SS is particularly implicated in the pathogenesis of gastroenteric infections [82,83]. These findings suggest that the coordinated activity of both secretion systems is essential for the full virulence expression of *A. caviae* V15^T^.

The isolation of multidrug-resistant *Aeromonas caviae* subsp. *aquatica* from domestic water storage tanks, containing many virulence factors, reveals a pressing and underrecognized vulnerability within Brazil’s water safety framework. Although the microbiological quality is guaranteed at the municipal treatment site by regulatory control, post-treatment storage, especially in residential reservoirs, remains a largely unregulated area vulnerable to microbial proliferation. Our data indicate that the existing guidelines recommending tank cleaning at six-month intervals may be inadequate, especially in tropical regions where elevated temperatures accelerate biofilm formation and support the persistence of antibiotic-resistant organisms. These findings underscore the urgent need to revise domestic water hygiene protocols and to incorporate environmental factors into risk assessment models for waterborne pathogens.

## 4. Conclusions

This study describes *Aeromonas caviae* subsp. *aquatica* subsp. nov., a novel multidrug-resistant subspecies isolated from a drinking water storage tank. The whole-genome sequencing of this strain revealed a variety of genes conferring resistance to antibiotics such as β-lactams, macrolides, quinolone, and multidrug efflux pumps. Furthermore, putative genes that could be involved in biofilm formation and pathogenicity mechanisms were identified. Monitoring *Aeromonas* in drinking water storage tanks is important as they are reservoirs of *Aeromonas* species and therefore may be important sources of human and animal infections. The genetic characterization of *Aeromonas* strains is essential for epidemiological surveillance and will advance our understanding of the unique genomic determinants underlying the pathogenicity of this emerging pathogen, while drinking water reservoirs have been identified as critical hotspots for antimicrobial resistance emergence. The findings underscore the critical need for the enhanced genomic surveillance of waterborne pathogens, public education campaigns about proper tank maintenance, and the development of innovative mitigation strategies, including advanced disinfection technologies, biofilm-targeted interventions, and real-time monitoring systems, to address this growing public health challenge at the human–animal–environment interface.

## Figures and Tables

**Table 1:** Antimicrobial susceptibility profiles of *A. caviae* isolates. (S, sensitive, or R, resistant.)

*A. caviae* Strain	AMK	AMP	ATM	CAZ	TGC	FEP	CIP	LEV	IPM	MEM	SXT	TZP
V15	4 (S)	≥64 (R)	32 (R)	≥64 (R)	≤0.5 (S)	128 (R)	≥4 (R)	≥8 (R)	8 (R)	≥16 (R)	≥150 (R)	≥128 (R)
FAHZZU2447	4 (S)	ND	32 (R)	≥128 (R)	≤0.5 (S)	128 (R)	16 (R)	ND	8 (R)	ND	≥150 (R)	≥128 (R)
211703	16 (S)	ND	≤1 (S)	≥64 (R)	≤0.5 (S)	ND	≥4 (R)	≥8 (R)	≥16 (R)	≥16 (R)	≥320 (R)	≥128 (R)
V123	4 (S)	≥64 (R)	32 (R)	≥8 (R)	≤0.5 (S)	128 (R)	≥4 (R)	≥8 (R)	8 (R)	≥16 (R)	≥150 (R)	≥128 (R)
V77	4 (S)	≥64 (R)	32 (R)	≥8 (R)	≤0.5 (S)	128 (R)	≥4 (R)	≥8 (R)	8 (R)	≥16 (R)	≥150 (R)	≥128 (R)
F10	4 (S)	≥64 (R)	≥8 (R)	≥8 (R)	≤0.5 (S)	≥8 (R)	≥4 (R)	≥8 (R)	8 (R)	≥16 (R)	≥8 (R)	≥8 (R)
AE27	4 (S)	≥64 (R)	≥8 (R)	≥4 (R)	≤0.5 (S)	≥8 (R)	≥4 (R)	≥4 (R)	8 (R)	≥16 (R)	≥8 (R)	≥8 (R)
V2	4 (S)	≥64 (R)	≥8 (R)	≥4 (R)	≤0.5 (S)	≥8 (R)	≥4 (R)	≥4 (R)	8 (R)	≥16 (R)	≥8 (R)	≥8 (R)
V51	16 (S)	≥64 (R)	≥4 (R)	≥4 (R)	≤0.5 (S)	≥8 (R)	≥4 (R)	≥4 (R)	8 (R)	≥16 (R)	≥8 (R)	≥8 (R)
V13	4 (S)	≥64 (R)	≥4 (R)	≥4 (R)	≤0.5 (S)	≥8 (R)	≥4 (R)	≥4 (R)	8 (R)	≥16 (R)	≥8 (R)	≥8 (R)
V7	4 (S)	≥64 (R)	≥4 (R)	≥4 (R)	≤0.5 (S)	≥8 (R)	≥4 (R)	≥4 (R)	8 (R)	≥16 (R)	≥8 (R)	≥8 (R)
V5	4 (S)	≥64 (R)	≥4 (R)	≥4 (R)	≤0.5 (S)	≥8 (R)	≥4 (R)	≥4 (R)	8 (R)	≥16 (R)	≥8 (R)	≥8 (R)
V44	16 (S)	≥64 (R)	≥4 (R)	≥4 (R)	≤0.5 (S)	≥8 (R)	≥4 (R)	≥4 (R)	8 (R)	≥16 (R)	≥8 (R)	≥8 (R)
AE21	16 (S)	≥64 (R)	≥4 (R)	≥4 (R)	≤0.5 (S)	≥8 (R)	≥4 (R)	≥4 (R)	8 (R)	≥16 (R)	≥8 (R)	≥8 (R)
AE33	4 (S)	≥64 (R)	≥4 (R)	≥4 (R)	≤0.5 (S)	≥8 (R)	≥4 (R)	≥4 (R)	8 (R)	≥16 (R)	≥8 (R)	≥8 (R)
F15	4 (S)	≥64 (R)	≥4 (R)	≥4 (R)	≤0.5 (S)	≥8 (R)	≥4 (R)	≥4 (R)	8 (R)	≥16 (R)	≥8 (R)	≥8 (R)
F17	4 (S)	≥64 (R)	≥4 (R)	≥4 (R)	≤0.5 (S)	≥8 (R)	≥4 (R)	≥4 (R)	8 (R)	≥16 (R)	≥8 (R)	≥8 (R)
F18	4 (S)	≥64 (R)	≥4 (R)	≥4 (R)	≤0.5 (S)	≥8 (R)	≥4 (R)	≥4 (R)	8 (R)	≥16 (R)	≥8 (R)	≥8 (R)
V1	16 (S)	≥64 (R)	≥4 (R)	≥4 (R)	≤0.5 (S)	≥8 (R)	≥4 (R)	≥4 (R)	8 (R)	≥16 (R)	≥8 (R)	≥8 (R)
V3	16 (S)	≥64 (R)	≥4 (R)	≥4 (R)	≤0.5 (S)	≥8 (R)	≥4 (R)	≥4 (R)	8 (R)	≥16 (R)	≥8 (R)	≥8 (R)
V4	16 (S)	≥64 (R)	≥4 (R)	≥4 (R)	≤0.5 (S)	≥8 (R)	≥4 (R)	≥4 (R)	8 (R)	≥16 (R)	≥8 (R)	≥8 (R)
V11	16 (S)	≥64 (R)	≥4 (R)	≥4 (R)	≤0.5 (S)	≥8 (R)	≥4 (R)	≥4 (R)	8 (R)	≥16 (R)	≥8 (R)	≥8 (R)
V87	4 (S)	≥64 (R)	≥4 (R)	≥4 (R)	≤0.5 (S)	≥8 (R)	≥4 (R)	≥4 (R)	8 (R)	≥16 (R)	≥8 (R)	≥8 (R)
V55	4 (S)	≥64 (R)	≥4 (R)	≥4 (R)	≤0.5 (S)	≥8 (R)	≥4 (R)	≥4 (R)	8 (R)	≥16 (R)	≥8 (R)	≥8 (R)
V23	4 (S)	≥64 (R)	≥4 (R)	≥4 (R)	≤0.5 (S)	≥8 (R)	≥4 (R)	≥4 (R)	8 (R)	≥16 (R)	≥8 (R)	≥8 (R)
V14	4 (S)	≥64 (R)	≥4 (R)	≥4 (R)	≤0.5 (S)	≥8 (R)	≥4 (R)	≥4 (R)	8 (R)	≥16 (R)	≥8 (R)	≥8 (R)
V61	4 (S)	≥64 (R)	≥4 (R)	≥4 (R)	≤0.5 (S)	≥8 (R)	≥4 (R)	≥4 (R)	8 (R)	≥16 (R)	≥8 (R)	≥8 (R)
V89	4 (S)	≥64 (R)	≥4 (R)	≥4 (R)	≤0.5 (S)	≥8 (R)	≥4 (R)	≥4 (R)	8 (R)	≥16 (R)	≥8 (R)	≥8 (R)
V42	4 (S)	≥64 (R)	≥4 (R)	≥4 (R)	≤0.5 (S)	≥8 (R)	≥4 (R)	≥4 (R)	8 (R)	≥16 (R)	≥8 (R)	≥8 (R)
V71	4 (S)	≥64 (R)	≥4 (R)	≥4 (R)	≤0.5 (S)	≥8 (R)	≥4 (R)	≥4 (R)	8 (R)	≥16 (R)	≥8 (R)	≥8 (R)
V78	4 (S)	≥64 (R)	≥4 (R)	≥4 (R)	≤0.5 (S)	≥8 (R)	≥4 (R)	≥4 (R)	8 (R)	≥16 (R)	≥8 (R)	≥8 (R)
V93	4 (S)	≥64 (R)	≥4 (R)	≥4 (R)	≤0.5 (S)	≥8 (R)	≥4 (R)	≥4 (R)	8 (R)	≥16 (R)	≥8 (R)	≥8 (R)
V75	4 (S)	≥64 (R)	≥4 (R)	≥4 (R)	≤0.5 (S)	≥8 (R)	≥4 (R)	≥4 (R)	8 (R)	≥16 (R)	≥8 (R)	≤4 (S)
V73	16 (S)	≥64 (R)	≥4 (R)	≥4 (R)	≤0.5 (S)	≥8 (R)	≥4 (R)	≥4 (R)	8 (R)	≥16 (R)	≥8 (R)	≤4 (S)
V32	16 (S)	≥64 (R)	≤1 (S)	≤1 (S)	≤0.5 (S)	≤1 (S)	≤0.25 (S)	≤2 (S)	≤0.25 (S)	≤1 (S)	≤1 (S)	≤4 (S)
V88	4 (S)	≥64 (R)	≤1 (S)	≤1 (S)	≤0.5 (S)	≤1 (S)	≤0.25 (S)	≤2 (S)	≤0.25 (S)	≤1 (S)	≤1 (S)	≤4 (S)
V49	4 (S)	≥64 (R)	≤1 (S)	≤1 (S)	≤0.5 (S)	≤1 (S)	≤0.25 (S)	≤1 (S)	≤0.25 (S)	≤1 (S)	≤1 (S)	≤4 (S)
V59	4 (S)	≥64 (R)	≤1 (S)	≤1 (S)	≤0.5 (S)	≤1 (S)	≤0.25 (S)	≤1 (S)	≤0.25 (S)	≤1 (S)	≤1 (S)	≤4 (S)
V53	16 (S)	≥64 (R)	≤1 (S)	≤1 (S)	≤0.5 (S)	≤1 (S)	≤0.25 (S)	≤1 (S)	≤0.25 (S)	≤1 (S)	≤1 (S)	≤4 (S)

A status of S, sensitive, or R, resistant, was assigned according to the BrCAST/EUCAST [46,47] and CLSI [48] clinical breakpoints. *Escherichia coli* ATCC 25922 and *Pseudomonas aeruginosa* ATCC 27853 served as quality controls. The *A. caviae* strains FAHZZU2447 [44] and 211703 [45] were used for comparison. AMK, amikacin; AMP, ampicillin; ATM, aztreonam; CAZ, ceftazidime; TGC, tigecycline; FEP, cefepime; CIP, ciprofloxacin; LEV, Levofloxacin; IPM, imipenem; MEM, meropenem; SXT, trimethoprim–sulfamethoxazole; TZP, piperacillin/tazobactam; ND, not determined.

**Table 2:** Pairwise comparisons of *A. caviae* V15^T^ and type strain genomes.

Subject Strain	dDDH (%)	Base Pairs	G + C Content (%)
*Aeromonas caviae* NCTC 12244	91.70	4,586,140	61.60
*Aeromonas hydrophila* subsp. *anaerogenes* CECT 4221	80.80	4,576,209	61.08
*Aeromonas sanarellii* LMG 24682	78.60	4,186,421	63.13
*Aeromonas taiwanensis* LMG 24683	76.80	4,230,588	62.83
*Aeromonas media* CECT 4232	71.50	4,467,324	61.15
*Aeromonas eucrenophila* CECT 4224	66.50	4,534,044	61.17
*Aeromonas hydrophila* ATCC 7966	62.70	4,744,448	61.55
*Aeromonas dhakensis* CIP 107500	61.80	4,711,264	61.78
*Aeromonas aquariorum* CECT 7289	61.60	4,677,943	61.95
*Aeromonas hydrophila* subsp. *ranae* CIP 107985	61.00	4,681,175	61.56
*Aeromonas trota* CECT 4255	50.40	4,332,624	60.07
*Aeromonas enteropelogenes* CECT 4487	48.90	4,461,928	59.70

**Table 3:** Virulence-associated genes found in *A. caviae* V15^T^.

Functions	Virulence Factors	Related Genes		
		*A. caviae* V15	*A. caviae* FAHZZU2447	*A. hydrophila* subsp. *hydrophila*
Adherence	Lateral flagela	*flgCEIJ*, *fliFGP*, *lafBCEFKSTUX*, *lfgABFGHKLMN*, *lfhAB*, *lfiEHIJMNQR*, *maf-5*	*flgCEIJ*, *fliFGP*, *lafBCEFKSTUX*, *lfgABFGHKLMN*, *lfhAB*, *lfiEHIJMNQR*, *maf-5*	-
	Mannose-sensitive hemagglutinin (Msh) pilus, type IV pili	*mshABCDEFGI1IJK* *LMNOPQ*	*mshABCDEFG1IJKLMNOP*	*mshBCDEFGI1IJLMNO*, *mshQ*
	Polar flagella	*cheA-2B-2R-3VWYZ*, *flaBHJ*, *flgABCDEFGHIJKLMN*, *flhABFG*, *fliAEFGHIJKLMNOPQR*, *flrABC*, *maf-1*, *motXY*, *pomA2AB2B*	*cheABRVWYZ*, *flaBHJ*, *flgABCDEFGHIJKLMN*, *flhABFG*, *fliAEFGHIJKLNOPQR*, *flrABC*, *maf-1*, *motXY*, *nueB*, *pomA2AB2B*	*cheA-2B-2R-3VWYZ*, *flaABGHJ*, *flgABCDEFGHIJKLMN*, *flhABF*, *fliAEFGHIJKLMNOPQR*, *flmDH*, *flrABC*, *maf-1-2*, *motXY*, *nueAB*, *pmA2AB2B*
	Tap type IV pili	*tapBCDFMNOPQTVW*, *tapY1*, *tppABCDEF*	*tapBCDFMNOPQTUVWY1*, *tppABCDE*	*tapABCDFMNOPQTUVW1*, *tppABEF*
Secretion system	T2SS	*exeABCDEFGHIJKLMN*	*exeABCDEFGHIJKLMN*	*exeABCDEFGHIJKLMN*, *tapD*
	T6SS	*atsDGHIJKLPQS*, *clpV1*, *dtU*, *hcp*, *vasHK/atsR*, *vgrG2G3*, *vipAB*	*atsD*	*atsABCDGHIJKLPQS*, *clpV1*, *dotU*, *hcp1*, *hcp*, *vasHK/atsR*, *vgrG1G2G3*, *vipAB*
Toxin	Hemolysin HlyA	*hlyA*	*hlyA*	*hlyA*

## Data Availability

The original contributions presented in this study are included in the article. Further inquiries can be directed to the corresponding author.

## Supplementary Materials

The following supporting information can be downloaded at https://www.mdpi.com/article/10.3390/microorganisms13040897/s1: Table S1: Virulence factors found in genome of strain V15^T^ and its respective genes and other *Aeromonas*.
